# Decline in Respiratory Functions in Hospitalized SARS-CoV-2 Infected Cancer Patients Following Cytotoxic Chemotherapy—An Additional Risk for Post-chemotherapy Complications

**DOI:** 10.3389/fmed.2022.835098

**Published:** 2022-03-10

**Authors:** Maha Ahmed Al-Mozaini, Mihyar Islam, Abu Shadat M. Noman, ATM Rezaul Karim, Walid A. Farhat, Herman Yeger, Syed S. Islam

**Affiliations:** ^1^Department of Infectious Disease and Immunity, King Faisal Specialist Hospital and Research Centre, Riyadh, Saudi Arabia; ^2^Department of Medicine, Parkview Hospital, Chittagong, Bangladesh; ^3^Department Biochemistry and Molecular Biology, University of Chittagong, Chittagong, Bangladesh; ^4^Department of Urology, University of Wisconsin School of Medicine and Public Health, Madison, WI, United States; ^5^Developmental and Stem Cell Biology, Peter Gilgan Centre for Research and Learning, The Hospital for Sick Children, Toronto, ON, Canada; ^6^Department of Molecular Oncology, King Faisal Specialist Hospital and Research Centre, Riyadh, Saudi Arabia; ^7^School of Medicine, Al-Faisal University, Riyadh, Saudi Arabia

**Keywords:** SARS-CoV-2, cancer, chemotherapy, DLCO, pulmonary impairment, HRCT (high resolution computed tomography)

## Abstract

**Background:**

Patients recovering from severe acute respiratory syndrome coronavirus 2 (SARS-CoV-2) infection demonstrate impaired lung function and those requiring chemotherapy after recovering from SARS-CoV-2 infection have yet to be explored. In this study, we sought to investigate the possible pulmonary functional changes during and after administering chemotherapy in patients with prior SARS-CoV-2 infection.

**Methods:**

In this study, a total of 37 SARS-CoV-2 infected patients with cancer who were discharged from hospital and received subsequent cytotoxic chemotherapy were enrolled and prospectively followed-up. The following parameters were prospectively measured before (P1), after first chemotherapy cycle (P2), and 10 weeks after the end of chemotherapy (P3), to assess their impact on respiratory complications in terms of diffusion capacity of the lungs for carbon monoxide (DLCO), forced expiratory volume in 1-s (FEV1), forced vital capacity (FVC), 6-min walking distance (6MWD) test and levels of key inflammatory markers.

**Results:**

All patients completed at least 2 cycles of chemotherapy without showing overt respiratory complications. Six patients (16%) complained about dyspnea during chemotherapy or at follow-up period. DLCO was significantly impaired during follow-up period [from P1 78 to P3 60% of predicted values; interquartile range (IQR) 55–89] and in 32 of 37 (86% of patients) from P1 to P2 (65% of predictive value; IQR 58–70; *p* < 0.001). Several patients experienced post-chemotherapy respiratory complications. As expected, all patients from control groups showed persistent improved pulmonary functions.

**Conclusion:**

The risk of pulmonary impairments due to cytotoxic chemotherapy in prior SARS-CoV-2 infected patients is linked to the loss of DLCO. Accordingly, we recommend that for patients with cancer requiring chemotherapy after recovering from prior SARS-CoV-2 infection, pulmonary tests to be performed routinely before and during chemotherapy treatment to monitor the pulmonary performance.

## Introduction

Patients with cancers are more likely to have disproportionately vulnerable and severe outcome from coronavirus disease 2019 (COVID-19) and poor prognosis in the case of immunocompromise as a result of chemotherapy. Many studies have reported the effects of anti-cancer treatment in patients with COVID-19 with conflicting results (1, 2). Emerging evidence demonstrate the symptoms and impairment of pulmonary function in patients who recover from severe COVID-19 infection (3–9). As COVID-19 symptoms and outcomes improve overtime, the long-term impact of respiratory impairments becomes of paramount importance. Most importantly, residual lung abnormalities have been detected in patients with severe COVID-19 infection after recovery (10–14). Cancer patients with blood, lung, or metastatic cancers were reported to have the highest frequency and severe outcomes (15, 16). Several recent reports demonstrated the severe impact of cancer treatment, such as chemotherapy, during the course of COVID-19 (1, 17–19). It is known that chemotherapy affects overall lung function, and many oncologists and societies recommend that the initiation or resumption of cytotoxic chemotherapy can be delayed until COVID-19 infected patients have completely recovered. Moreover, many cancer patients with advanced stage tumor progression require urgent chemotherapy-based treatment. Therefore, it is pivotal to investigate whether administering chemotherapy can cause the additional risk of pulmonary impairments, performance, and tolerance in cancer patients with prior severe COVID-19 infection.

This study was aimed to prospectively and systematically evaluate the possible association of chemotherapy with pulmonary functions on patients with cancer scheduled for chemotherapy treatment after recovery from COVID-19. The primary focus was to identify the changes in lung function during chemotherapy and any potential association with post therapy respiratory complications.

## Patients and Methods

This prospective cohort study population consisted of 37 cancer patients with a history of recent severe acute respiratory syndrome coronavirus 2 (SARS-CoV-2) infection who received post-infection multi-agent chemotherapies from June 12, 2020 to June 15, 2021. All patients were throat swabbed and screened for SARS-CoV-2 RNA by real-time reverse transcriptase PCR (RT-PCR) before administering chemotherapy (negative for viral RNA). Patients with a history of the following underlying conditions, i.e., hypertension, cardiovascular disease, diabetes, chronic lung disease, chronic liver and renal disease, asthma or short and long history of smoking, and chronic obstructive pulmonary disease (COPD) were excluded from the study. In addition, during hospitalization, patients who required intubation or invasive mechanical ventilation were excluded from the study given that the potential for the consequences of mechanical ventilation may influence the factors under investigation. As control group, a cohort of patients with cancer who have been treated 2–6 months before COVID-19 infection (*n* = 38), a cohort of patients without cancer (*n* = 37) and a cohort of patients with cancer but no history of COVID-19 (*n* = 10) were included in the study. This study was approved by the ethics committee of Bangladesh Medical Research Council (BMDC) and Park View Hospital [study # 2021-2023/62(1-20)]. Written informed consent was obtained from all participants. All research was performed in accordance with the relevant ethical guidelines and regulations.

### Lung Functions Assessment

Patients demographics, cancer details, and cancer treatment information were collected. Patients had received multi-agent chemotherapies or a combination of chemotherapy and targeted therapy followed by granulocyte colony-stimulating factor (G-CSF) support. All participants were assessed before starting chemotherapy (P1). Assessment was repeated after first chemotherapy cycle (P2) and 10 weeks after the end of chemotherapy (P3). During and after chemotherapy, patients were interviewed and following examinations were performed: the measurements of diffusing capacity of the lungs for carbon monoxide (DLCO), forced vital capacity (FVC), functional residual capacity as FEV, residual value (RV), total lung capacity (TLC), and 6-min walking distance (6-MWD) test. DLCO was measured using as a single-breath test and adjusted for hemoglobin, after capillary blood gas analysis. The baseline laboratory values of serum creatinine, D-dimers, ferritin, and inflammatory maker interleukin-6 (IL-6) were assessed in each follow-up time points. All pulmonary function test measurements were expressed and presented as the percentage (%) of predicted normal values. Diffusion deficit was considered with a DLCO value less than (<80%) of the predicted value (11, 20).

### Chest CT Scan Examinations

For chest, non-contrast enhanced high-resolution CT (HRCT) examination, individuals were placed in a supine position with breath holding following inspiration (Definition prospective, Siemens, Germany). Patients were scanned using a 124-slice section multidetector CT scanner with the following parameters of 10 and 0.75 mm collimation. The HRCT scans were conducted once at the beginning of the treatment. The resulting images were visualized and image archiving using a communication system with standard lung (width 1,600 Hounsfield Unit [HU], level −400 HU) and mediastinal (width 400 HU and level 50 HU) window settings. Chest computed tomographic scans were evaluated by two highly experienced practitioners, a radiologist and a pulmonologist, and images were reviewed in a blind folded manner regarding the clinical and functional condition of the patients for the analysis of changes in lung function.

### 6MWD Test

A 6MWD distance test was performed in room air according to the American Thoracic Society (ATS) guidelines (21). Each patient, at each follow-up time point, was asked to walk on a flat, hard surface ground as quickly as possible in a period of 6 min. The results were expressed as “meters” and the percentage of predicted values calculated using a method described previously (22).

### Statistical Analysis

All statistical analyses were performed using R (version 4.0.3; 2020-10-10) statistical software and “ggplot2” package for generating graphs. Continuous variables were expressed as median and interquartile range (IQR) and differences were assessed using two-sample *t*-test, Welch's two sample *t*-test. For group comparison of continuous data, Mann–Whitney *U*-test, Kruskal–Wallis test, and repeated measure ANOVA were applied if appropriate. The values of *p* < 0.05 were considered statistically significant.

## Results

**Figure 1:**
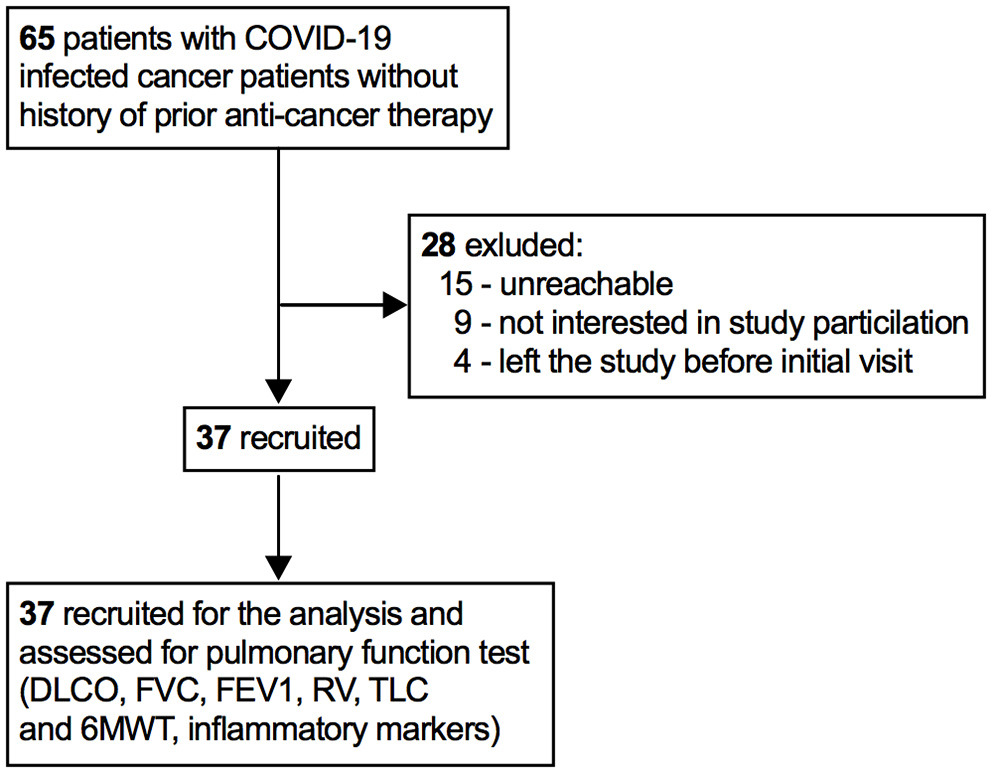
Patients recruitment profile. DLCO, lung diffusion for carbon monoxide; FVC, forced vital capacity; FEV1, forced expiratory volume in 1 second; RV, residual volume; TLC, total lung capacity; 6MWT, 6-minute walking time.

In total, 37 patients with cancer who recovered from SARS-CoV-2 infection were recruited to the study between June 12, 2020 and June 15, 2021 (Figure 1). There were 23 (62%) men and 14 (38%) women. Patients never received any anti-cancer treatment before being infected with SARS-CoV-2. The median age was 54 years (IQR 42–72) and the median body mass index (BMI) was 28.5 Kg/m^2^ (IQR 25.2–30.7 Kg/m^2^). Six (16%) patients reported dyspnea and cough during hospital stay. The average length of hospital stay was 21 days (IQR 13–29). Table 1 summarizes the patients demographic and clinical characteristics. Before administering chemotherapy, all patients had tested negative for SARS-CoV-2, however, several patients particularly age between 62 and 75 years had post-COVID-19 symptoms, such as, difficulty of breathing, sleep problems, and cough.

**Table 1:** Characteristics of patients with cancer who were infected with SARS-CoV-2 before starting anticancer treatment.

**Patients characteristics**	**Patients (*n* = 37)**
Age, years	54 (42–72)
**Sex, n (%)**	
Male	23 (62%)
Female	14 (38%)
^a^BMI (kg/m^2^)	28.5 (25.2–30.7)
**Hospitalization status, n (range)**	
Length of hospital stay (days)	21 (13–29)
Length of ICU stay (days)	16 (11–21)
**Supplemental oxygen requirement**	
Nasal canula	23 (62%)
High flow nasal canula	13 (35%)
**Disease severity**	
Mild	9 (24%)
Severe/Critical	23 (62%)
**Clinical manifestation of COVID-19**	
Fever	16 (43%)
Cough	23 (62%)
Fatigue	9 (24%)
Chest tightness	7 (19%)
Shortness of breath	17 (46%)
Dyspnea	9 (24%)
Headache	5 (14%)
**Types of cancer**	
Breast	9 (24%)
Lung	7 (19%)
Colon	4 (11%)
Head and neck	3 (8%)
Rectal	3 (8%)
Ovary	5 (14%)
Esophagus	2 (5%)
Cervical	2 (5%)
Gastric	2 (5%)

*Data are median (interquartile range [IQR]) or n (%)*.

a *BMI-body mass index*.

Patients received treatment for SARS-CoV-2 infection, such as antiviral treatment (7/37, 19%), antibiotic treatment (6/37, 16%), and immunomodulatory treatment (3/37, 8%) during hospital stay. Four (11%) patients received oxygen inhalation through nasal cannula and none of the patients received invasive mechanical ventilation or intensive care unit (ICU). None of the patients received chemotherapy or targeted therapy or radiotherapy before being infected with SARS-CoV-2. All patients survived up until the end of the follow-up period.

**Figure 2:**
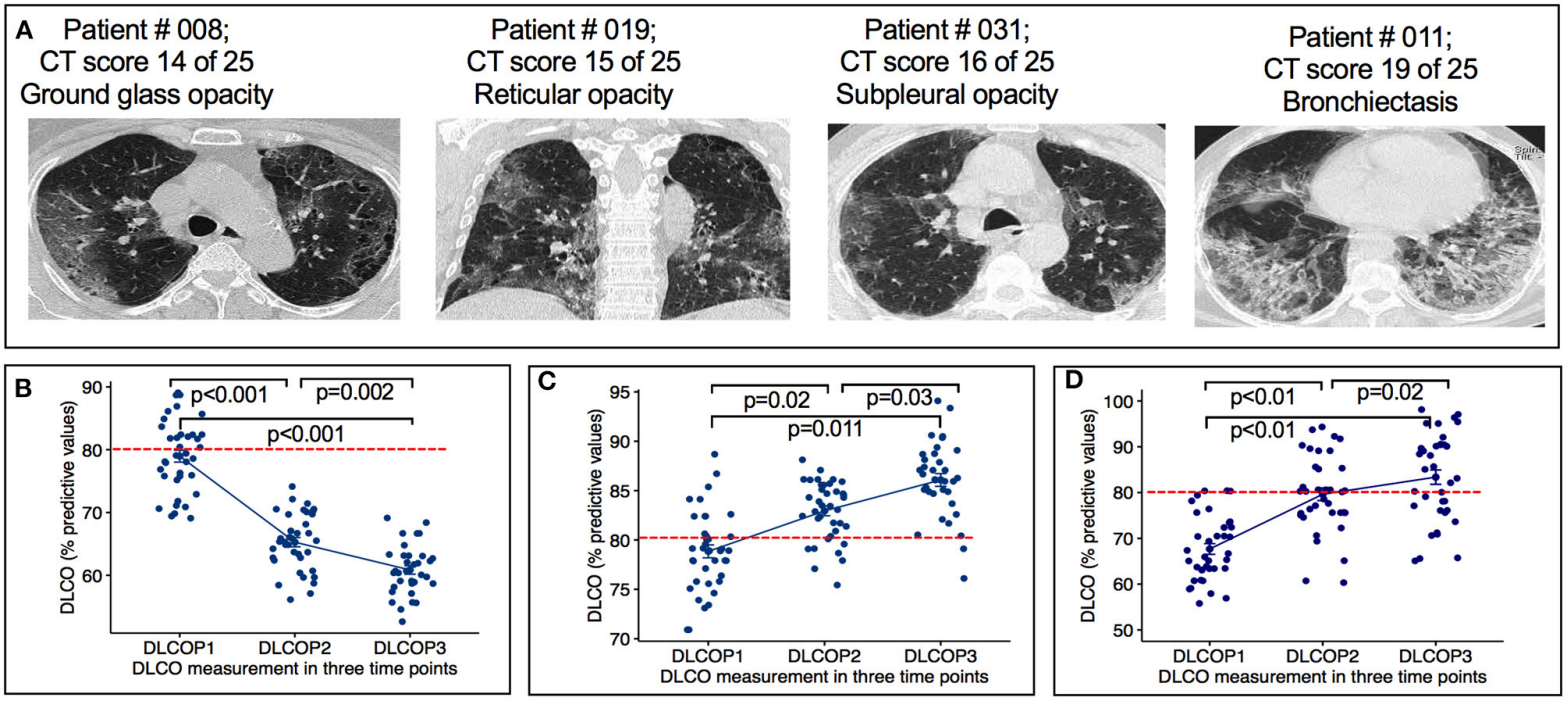
Radiographic features of HRCT images and DLCO measurement from COVID-19 infected cancer patients. **(A)** Representative images showing ground glass opacity, reticular opacity, subpleural opacity and bronchiectasis of indicated patients. **(B)** Figure show the changes in lung diffusion for carbon monoxide (DLCO) at each time point. SARS-CoV-2 infected cancer patients treated with chemotherapy before (P1), after first cycle of chemotherapy (P2) and 10 weeks after the end of chemotherapy (P3). *P* < 0.0001: changes of DLCO from P1 to P2, *p* = 0.002: Changes of DLCO from P2 to P3. **(C)** Graph show the change of DLCO of cancer patients treated with chemotherapy 2–6 months before SARS-CoV-2 infection at three time points. Changes of DLCO from P1 to P2, *p* = 0.02: changes of DLCO from P2 to P3, *p* = 0.03. Horizontal broken red line indicates the normal cutoff of 80%. **(D)** Graph show the change of DLCO of non-cancer SARS-CoV-2 infection only patients at three time points. Changes of DLCO from P1 to P2, *p* < 0.01: changes of DLCO from P2 to P3, *p* = 0.02. Horizontal broken red line indicates the normal cutoff of 80% predicted. DLCOP1, measurement of DLCO before administering chemotherapy; DLCOP2, measurement of DLCO after first chemotherapy cycle, DLCOP3, measurement of DLCO ten weeks after the end of chemotherapy. Horizontal broken red line indicates the normal cutoff of 80% predicted.

All patients had chest HRTC scans at initial hospital admission. Patients demonstrated a wide array of CT scans abnormalities, predominantly 21 (57%) showed the existence of reticular opacity, 27 (78%) showed the presence of ground glass opacity, 19 (51%) subpleural opacity, and 11 (30%) bronchiectasis (Figure 2A).

Among all patients, breast cancer was the most abundantly detected cancer (9/37, 24%), followed by lung (7/37, 19%) and ovarian cancer (5/37, 13%). Fifteen (38%) patients had advanced stage IV disease with distant metastasis. All patients successfully completed multi-agent chemotherapy or a combination of chemotherapy. Patients were treated with different chemotherapeutic agents based on the type of cancers and TNM (Tumor Nodes Metastasis) stages. Patients with lung cancer received GP (gemcitabine and cisplatin); patients with breast cancer received GP and docetaxel, docetaxel and capecitabine, and docetaxel alone; patients with colon cancer received capecitabine and XELOX (oxaliplatin and capecitabine); patients with head and neck cancer received DP (docetaxel and cisplatin) and cisplatin; patients with rectal cancer received FOLFOX (oxaliplatin, 5-fluorouracil, and leucovorin) and DC (docetaxel and carboplatin); patients with ovarian cancer received TP (paclitaxel and cisplatin); patients with esophagus cancer received TP (paclitaxel and cisplatin); patients with cervical cancer received DP (docetaxel and cisplatin); and patients with gastric cancer received XELOX (oxaliplatin and capecitabine) therapy regiments. The chemotherapeutic outcomes were assessed at the completion of therapy. Fifteen (41%) patients were identified to have a partial response, meaning further treatment would probably be required to attempt a full cure, 10 (27%) patients were considered as pathological responsive to therapy with the expectation of long-term disease-free survival and overall survival, and 12 (32%) patients were considered to have stable disease. The time between hospital discharge and the first administration of chemotherapy varied between patients. The median time was 55 days (IQR 48–90).

Six (16%) patients reported dyspnea during follow-up period. All 37 patients demonstrated significant decrease in diffusing capacity of the lungs for carbon monoxide (DLCO) from P1 (78%, IQR 67–89) to P3 (60%; IQR 55–69). Of 37 patients, 32 (86%) patients demonstrated significant decrease in DLCO predicted values from P1 (78%, IQR 67–89) to P2 (65%; IQR 58–70) (*p* < 0.001) (Figure 2B). At P1, all patients had a DLCO predicted value above 70%, and 40% patients tested above 80% predicted DLCO value, whereas, in P2 and P3, none of the patients achieved this result. At P2, the DLCO values continued to decrease and persisted until P3 (Figure 2B, Table 2). The decrease of predicted DLCO from P1 (78%, IQR 67–89) to P3 (60%; IQR 55–69) was also seen in patients (Figure 2B, *p* < 0.001). In addition, the DLCO/Va was decreased from P1 (81%; IQR 71–92) to P2 (73%, IQR 69–82) of the predicted value (*p* < 0.01) and further declined from P2 to P3 (67% IQR 64–81) of the predicted value (*p* = 0.03) (Table 2). Median FVC was 78% of predicted (IQR 71–82) at P1 (*p* < 0.001), slightly decreased at P2 and P3 (Table 2; *p* = 0.25). Median FEV1 was 81% of predicted at P1 (IQR 76–85; *p* = 0.40) and stayed at 79% predicted at P2 and P3 (*p* = 0.53), respectively. RV and TLC were 82% (IQR 75–87; *p* = 0.03; IQR 76–86; *p* < 0.001) of predicted at P1 and continued to slightly decline at P2 and P3 (Table 2). The median 6MWD test (% predicted) at P1 was 497 (IQR 475–509) and declined from P1 to P2 (439; IQR: 422–478; *p* = 0.03) with a further decline from P2 to P3 (422; IQR 410–438; *p* < 0.01) (Table 2). During chemotherapy, respiratory function parameters DLCO (*p* < 0.001), FVC (*p* < 0.05), and 6MWD (*p* = 0.03) were correlated to respiratory complications and may be these factors affecting the development of respiratory impairments (Table 3). During the treatment period, several patients showed respiratory complications. Seven patients reported acute respiratory failure requiring intubation or non-invasive ventilation, 4 cases reported pneumonia, and 1 case of pulmonary edema (deposit of fluids in lung). These complications were solely observed in the study patient's cohort.

**Table 2:** Lung function values before the beginning of chemotherapy and during chemotherapy treatment follow-up.

**Parameters**	**P1 (*n* = 37)**	**P2 (*n* = 34)**	**P3 (*n* = 34)**	***P*-value^**h**^**	***P*-value^**i**^**
DLCO^a^ (% predicted)	78 (67–89)	65 (58–70)	60 (55–69)	<0.001	0.002
DLCO/Va^b^ (% predicted)	81 (71–92)	73 (69–82)	67 (64–81)	<0.01	0.03
FVC^c^ (% predicted)	78 (71–82)	73 (69–82)	72 (70–82)	<0.001	0.25
FEV1^d^ (% predicted)	81 (76–85)	79 (77–84)	79 (76–85)	0.40	0.53
RV^e^ (% predicted)	82 (75–87)	77 (73–81)	74 (74–82)	0.03	<0.001
TLC^f^ (% predicted)	82 (76–86)	81 (75–86)	81 (75–83)	0.006	0.38
6MWD^g^ (meter)	497 (475–509)	439 (422–478)	422 (410–438)	0.03	0.01
6MWD^g^ (% predicted)	97 (77–106)	79 (70–91)	64 (61–81)	0.01	0.03

*Data are median and IQR; P1, measurement before chemotherapy; P2, measurements after the first cycle of chemotherapy; P3, measurements 5 weeks after chemotherapy*;

a *DLCO, Diffusing lung capacity for carbon monoxide*;

b *Va, alveolar volume*;

c *FVC, Forced vital capacity*;

d *FEV1, Forced expiratory volume in 1-second*;

e *RV, Residual volume*;

f *TLC, Total lung capacity*;

g *6MWT, Six-min walking time*;

h *P2 vs. P1*;

i *P3 vs. P2*.

**Table 3:** The correlation of pulmonary function and complications in patients with cancer who underwent anti-cancer therapy.

	**SARS-CoV-2 infected cancer patients' severity during chemotherapy**		***P*-value**
	**Complication (*n* = 25)**	**Not complication (*n* = 12)**	
Male/Female	20/5	9/3	0.21
DLCO^a^ (% predicted)	68 (62–73)	71 (69–81)	<0.001
FVC^b^ (% predicted)	73 (69–78)	76 (72–81)	<0.05
FEV1^c^ (% predicted)	78 (76–83)	81 (78–89)	0.07
RV^d^ (% predicted)	76 (72–82)	76 (70–82)	0.15
TLC^e^ (% predicted)	79 (77–85)	81 (77–84)	0.35
6 MWD^f^ (meter)	491 (462–501)	499 (479–516)	0.03
6 MWD^f^ (% predicted)	75 (63–89)	83 (73–98)	0.02

*Data are median and (IQR)*,

a *DLCO, Diffusing lung capacity for carbon monoxide*;

b *FVC, Forced vital capacity*;

c *FEV1, Forced expiratory volume in 1-second*;

d *RV, Residual volume*;

e *TLC, Total lung capacity*;

f *6MWD, Six-min walking distance*.

As a control group, we analyzed DLCO values in 2-, 3-, and 4-months interval from a group of patients with cancer (*n* = 38), who had been treated with similar chemotherapy regimens 2–6 months prior COVID-19 infection. At P1, all patients continued to increase and outperform the pulmonary function as measured by DLCO values (Figure 2C; P1 to P2 *p* = 0.02 and P2 to P3 *p* = 0.03). Median FVC was 78% of predicted (71–82) at P1 (*p* < 0.001), increased at P2 and P3 (Table 4; *p* = 0.03). All other parameters, forced expiratory volume in 1-s (FEV1), residual volume (RV), total lung capacity (TLC), and 6MWD were increased gradually (Table 4). In addition, we used a cohort of COVID-19 only patients (*n* = 37) group who had no history of cancer otherwise and measured the DLCO values at 2-, 4-, and 6-months interval (Figure 2D). Table 4 summarizes the pulmonary parameters for these patients. Finally, we measured and analyzed the DLCO values from a group of patient (*n* = 13) who were diagnosed with cancer and had chemotherapy treatment but no history of COVID-19 infection. In this group, as expected, we observed that patients with lung cancer (*n* = 6) who received chemotherapy had impaired DLCO values, while no substantial pulmonary impairments were noted in breast (*n* = 3) and ovarian cancer (*n* = 4) patients after receiving chemotherapy. We did not present the details of these patients due to lack of sufficient number of patients. Taking all together, our results confirmed that patients with cancer who were undergoing chemotherapy treatment with prior COVID-19 experienced pulmonary impairments.

**Table 4:** Lung function values of patients who received chemotherapy 2–6 months before severe acute respiratory syndrome coronavirus 2 (SARS-CoV-2) infection.

**Parameters**	**2-months (*n* = 37)**	**3-momths (*n* = 34)**	**4-months (*n* = 34)**	***P*-value^**g**^**	***P*-value^**h**^**
DLCO^a^ (% predicted)	73 (65–87)	76 (64–90)	81 (75–96)	0.02	0.03
FVC^b^ (% predicted)	78 (71–82)	81 (72–85)	84 (77–92)	<0.001	0.03
FEV1^c^ (% predicted)	77 (69–83)	81(79–86)	82 (79–97)	0.40	0.53
RV^d^ (% predicted)	88 (76–92)	89 (83–93)	89 (84–101)	0.06	0.09
TLC^e^ (% predicted)	86 (73–91)	93 (85–102)	93 (82–103)	0.06	0.38
6MWD^f^ (meter)	489 (455–501)	502 (462–514)	502 (478–527)	0.03	0.07
6MWD^f^ (% predicted)	78 (69–85)	92 (84–101)	99 (88–103)	0.01	0.02
**Lung function values of non-cancer SARS-CoV-2 infected patients**					
DLCO^a^ (% predicted)	75 (64–86)	79 (67–91)	86 (74–98)	0.02	<0.01
FVC^b^ (% predicted)	79 (72–84)	81(73–86)	86 (76–97)	0.03	0.003
FEV1^c^ (% predicted)	77 (69–83)	83 (77–87)	89 (81–98)	0.04	0.02
RV^d^ (% predicted)	88 (74–91)	90 (82–98)	92 (86–101)	0.06	0.09
TLC^e^ (% predicted)	85 (73–92)	92 (80–101)	94 (82–107)	0.03	0.08
6MWD^f^ (meter)	491 (455–505)	504 (462–517)	509 (478–527)	0.03	0.04
6MWD^f^ (% predicted)	79 (71–85)	91 (87–100)	100 (91–105)	0.001	0.03

*Data are median and IQR*;

a *DLCO, Diffusing lung capacity for carbon monoxide*;

b *FVC, Forced vital capacity*;

c *FEV1, Forced expiratory volume in 1-second*;

d *RV, Residual volume*;

e *TLC, Total lung capacity*;

f *6MWD, Six-min walking distance*;

g *3-months vs. 2-months*;

h *4-months vs. 3-months*.

**Figure 3:**
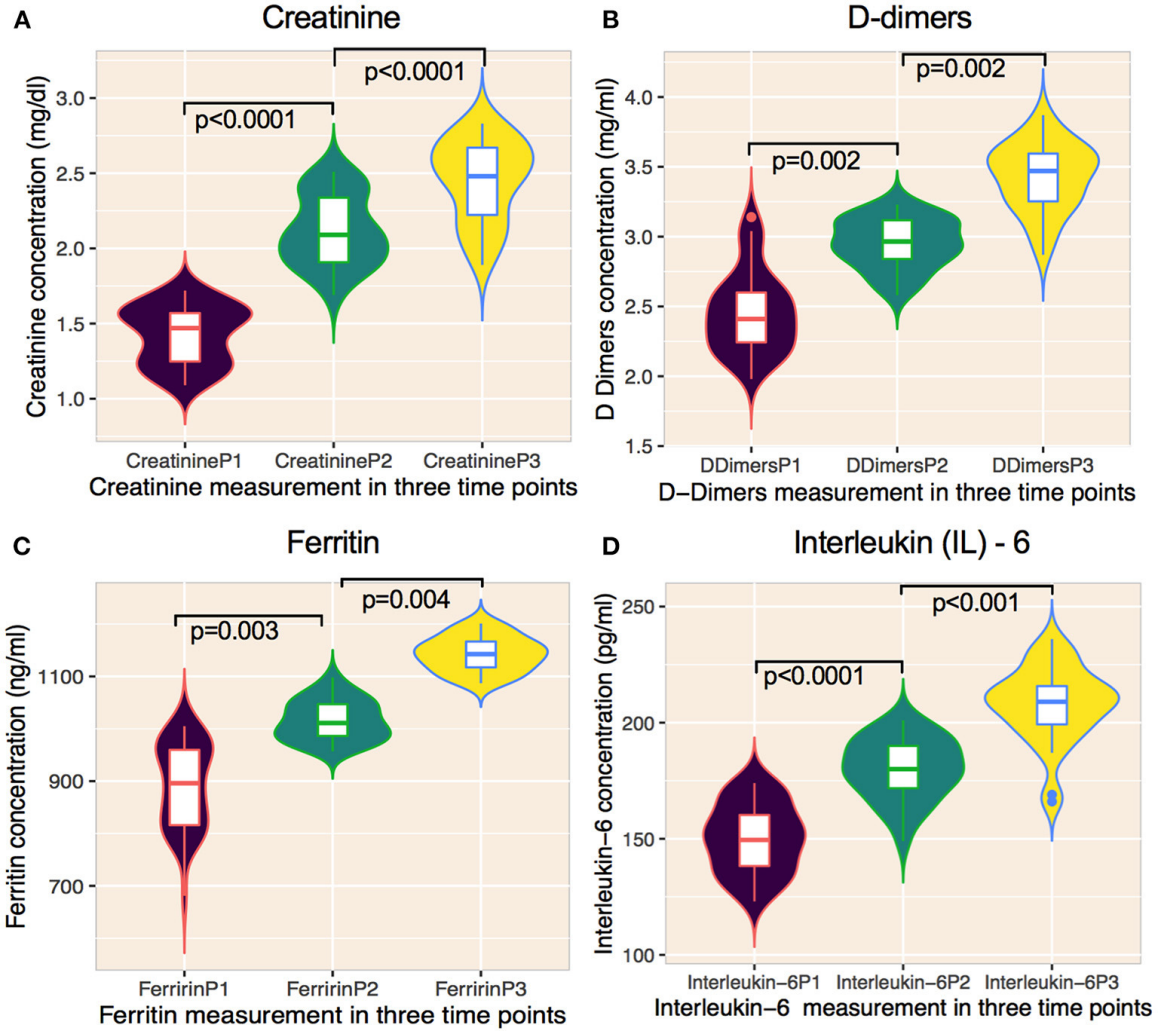
Changes of laboratory features on cancer patients undergoing cytotoxic chemotherapy on prior COVID-19 infected patients. **(A–D)** Graphs showing the temporal changes in creatinine and inflammatory markers. **(A)** creatinine, **(B)** D-dimers, **(C)** ferritin, and **(D)** interleukin-6) of cancer patients infected with SARS-CoV-2 and subsequently treated with anti-cancer therapy. Data are median (IQR). Solid lines within violins represents median, 25% percentile, and 75% percentile. Violins show min-max ranges. The Mann-Whitney U test was used for calculating *p* values. P1, measurement before administering chemotherapy; P2, measurement after first chemotherapy cycle, P3, measurement 10 weeks after the end of chemotherapy.

Significant changes in creatinine, D-dimers, ferritin, and IL-6 were noted from P1 to P3 (Figures 3A–D, Table 5) compared with those who received chemotherapy 2–6 months prior to virus infection and non-cancer patients (Table 6). Changes in serum hemoglobin (Hb), erythrocyte sedimentation rate (ESR), and C-reactive protein (CRP) are shown in Table 5. Hemoglobin levels declined throughout the treatment period particularly from P1 (13.6 g/dl; IQR 10.6–17.1) to P2 (11.1 g/dl; IQR 9.7–15.5), and P2 to P3 (10.8 g/dl; IQR 8.4–15.7). ESR continued to be increased from P1 (53.1 mm/dl; IQR 47.4–62.7) to P2 (61.4 mm/dl; IQR 58.1–65.3) to P3 (68.1 mm/dl; IQR 58.7–70.7). Similarly, CRP tended to gradually increase from P1 (171.1 mg/dl; IQR: 159.2–179.5) to P2 (187.7 mg/dl; IQR 177.2–193.4) to P3 (201.5 mg/dl; IQR 189.6–215.1).

**Table 5:** Baseline creatinine, and inflammatory markers, D-dimers, ferritin, and interleukin-6 (IL-6) of patients who were undergoing chemotherapy after recovery from SARS-CoV-2 infection.

**Parameters**	**P1 (*n* = 34)**	**P2 (*n* = 30)**	**P3 (*n* = 30)**	***P*-value^**a**^**	***P*-value^**b**^**
Creatinine (mg/dl)	1.47 (1.09–1.72)	2.09 (1.9–2.51)	2.48 (2.22–2.83)	<0.0001	<0.0001
D-dimers (μg/ml)	2.41 (2.25–3.14)	2.96 (2.84–3.23)	3.47 (3.25–3.87)	0.002	0.002
Ferritin (ng/ml)	852 (789–1,005)	1,001 (957–1,097)	1,104 (1,087–1,201)	0.003	0.004
IL-6 (pg/ml)	151 (123–174)	174 (159–201)	189 (166–219)	<0.0001	<0.001
Hb (g/dL)	13.6 (10.6–17.1)	11.1 (9.7–15.5)	10.8 (8.4–15.7)	0.003	0.01
ESR (mm/hr)	53.1 (47.4–62.7)	61.4 (58.1–65.3)	68.1 (58.7–70.7)	0.001	<0.0001
CRP (mg/L)	171.1 (159.2–179.5)	187.7 (177.2–193.4)	201.5 (189.6–215.1)	0.002	<0.0001

*Data are median and IQR; P1, measurement before chemotherapy; P2, measurements after first cycle of chemotherapy; P3, measurements 5 weeks after the end of chemotherapy; IL-6, Interleukin-6; Hb, hemoglobin; ESR, erythrocyte sedimentation rate; CRP, C-reactive protein*.

a *p: P2 vs. P1*;

b *P: P3 vs. P2*.

**Table 6:** Baseline creatinine, and inflammatory markers, D-dimers, ferritin and IL-6 of patients with cancer who underwent chemotherapy before SARS-CoV-2 infection.

**Laboratory markers**	**2-months (*n* = 21)**	**3-months (*n* = 20)**	**4-months (*n* = 17)**	***P*-value^**a**^**	***P*-value^**b**^**
Creatinine	1.21 (0.97–1.57)	1.07 (0.79–1.39)	0.79 (0.59–1.05)	<0.001	0.034
D-dimers	1.98 (1.25–2.05)	1.05 (0.87–1.58)	0.69 (0.58–1.14)	<0.001	0.014
Ferritin	345 (298–405)	209 (148–317)	121 (97–169)	0.44	0.027
Interleukin-6	125 (98–189)	92 (69–137)	41 (29–78)	<0.05	0.031
**Baseline creatinine, and inflammatory markers, D-dimers, ferritin and IL-6 of non-cancer SARS-CoV-2 infected patients after discharged from hospital**.					
Creatinine	1.01 (0.92–1.11)	0.97 (0.93–1.13)	0.94 (0.89–1.05)	0.02	0.03
D-dimers	1.78 (1.33–2.11)	1.26 (0.86–1.38)	1.09 (0.88–1.27)	<0.01	0.014
Ferritin	363 (277–417)	371 (271–433)	401 (307–481)	0.04	0.03
Interleukin-6	92 (78–109)	93 (79–117)	94 (73–113)	0.15	0.04

*Data are median and IQR*;

a *2-months vs. 1-months*;

b *3-months vs. 2-months*.

## Discussion

Recent evidence suggests that residual lung abnormalities have been found in patients with severe SARS-CoV-2 months after recovered from the disease (11–14). However, these abnormalities have not been systematically evaluated for patients with cancer who were infected with SARS-CoV-2 before the administration of chemotherapy and therefore remains to be determined. Our study revealed a significant reduction in pulmonary function in all patients who were recently treated with chemotherapy administered after discharged from hospital with prior SARS-CoV-2 infection. The changes in pulmonary function occurred during the treatment period and persisted at least to the end of treatment. These findings amplify the importance of practicing urgent need of care for patients with cancer who are scheduled to resume anti-cancer treatment or undergo first time chemotherapy after recovering from COVID-19.

Potential respiratory impairments due to COVID-19 infection in recovered patients receiving chemotherapy is a major debate and has been receiving greater attention from oncologists, public health professionals, and professional societies. Many studies have reported that patients with severe and/or critical SARS-CoV-2 infection demonstrate a wide array of pulmonary impairments, such as pulmonary fibrosis, bronchiectasis, pulmonary and systemic disease (23), and multiple organ dysfunction (24). Changes of respiratory functions after recovering from COVID-19 infection are clinically important because COVID-19 infection not only increases the risk of severe outcomes but can also have an impact on post-COVID-19 cancer therapy for clinically vulnerable patients with cancer. Residual lung abnormalities have been found in patients with SARS-CoV-2 after recovery (25, 26). We previously explored the impact of severe COVID-19 and the role of viral load in cancer and non-cancer patients (27). Pulmonary impairment concerns from a prior infection and are not only limited to COVID-19 infection. COPD is considered to be an important risk factor particularly for patients with lung cancer (28). It was shown that the decline in pulmonary functions in patients with breast cancer who are receiving dose-dense chemotherapy (DDS) was associated with a significant reduction in DLCO (29). It was reported that patients receiving chemotherapy (mainly cisplatin) for lung cancer showed improvements in spirometric performance, however, concomitant with the reduction in lung diffusion capacity (30). Many recent reports illustrate the severe impact of cancer treatment on recovered and surviving COVID-19 patients (1, 17–19) with clear contrast to patients who are not receiving chemotherapy (19, 31). These studies mainly restricted to address whether patients with cancer who received chemotherapy prior COVID-19 infection, could predict an increased rate of severe or critical COVID-19 events. To date, no studies have shown whether chemotherapy would make cancer patients with prior COVID-19 infection more vulnerable to pulmonary function impairment. To address the outstanding unknown consequences, we assessed the pulmonary function on patients with cancer who were infected with COVID-19 and underwent chemotherapy treatment. Our study demonstrates that administering chemotherapy to these patients is associated with increased and persistent pulmonary impairments assessed by the reduction of DLCO, pulmonary physiology, and exercise capacity. The decrease of DLCO after chemotherapy (P2) was found in 86% of patients, however, all patients maintained DLCO >60% of the predicted value. If we consider that changes of DLCO represent subclinical damage/impact, the degree of such damage/impact may be due to the loss of DLCO, further influencing the risk of respiratory complications. As this study was designed to assess the relationship between response to chemotherapy and respiratory function of recovered COVID-19 infected patients, we found that all patients had lower post-chemotherapy DLCO predicted value. Moreover, all patients recruited in our study had never smoked, and no patient had a history of hypertension, diabetes, asthma, or COPD as these factors may influence the lung function and DLCO results. We therefore speculate that severe COVID-19 infection was the primary cause for decreased pulmonary function, and that chemotherapy influenced further reduction in respiratory function, which may be an additional risk factor for patients with cancer during and after administering chemotherapy treatment.

The question remained to be determined is when do DLCO/impaired lung functions changes occur and how long do they last? As all patients included in this study already had some form of impaired pulmonary functions due to COVID-19, administering chemotherapy probably influenced the deterioration in lung function and changes in DLCO, and may last longer after the completion of chemotherapy. We therefore suggest designing a future study to assess the long-term impact of cytotoxic chemotherapy on the pulmonary functions of recovered patients with COVID-19 which could explain the absence of pulmonary complications.

Evidence about pulmonary function test among SARS-CoV-2 infected patients with cancer who are expected to start anti-cancer therapy is currently not in practice. Mo et al. (26) reported that nearly a month after hospital discharge, regardless of the degree of SARS-CoV-2 severity, no significance differences in FVC were observed. Fumagalli et al. (32) reported a significant reduction in FVC 6 weeks after hospital discharge. In both cases, the sample size was significantly smaller. Wu et al. (12) reported that only COVID-19 patients without a history of cancer, the median FVC was 92% of predicted at 3 months after recovery from COVID-19, and gradually increased at 6 and 9 months, respectively, suggesting that declining lung impairment was observed primarily for severe COVID-19 and that lung functions normalized over time. In our case, we have noticed that the median FVC was 78% of predicted before the start of the first anti-cancer treatment cycle and gradually decreased at first and second cycle. Our findings are consistent with a previous COVID-19 follow-up study and we identified that cancer patients with prior COVID-19 who were undergoing anti-cancer therapy predicted impaired FVC during and after anti-cancer treatment.

Our study has several limitations. First, the number of cases in this study is relatively small, which may lead to some biases in the results. Larger sample size cohorts are needed to confirm our results. Second, the duration of the follow-up is relatively shorter and it may take a longer period of time to determine the complete pulmonary functions caused by either COVID-19 infection and/or chemotherapy. Third, as most chemotherapy drugs are toxic to lung, and we are aware that the results may lead to some extent of bias of pulmonary impairments. Fourth, we acknowledge that the CT scans, patient's hemoglobin levels, and the minimum oxygen saturation levels at the end of 6MWD is an integral part before beginning or during chemotherapy. Despite our full efforts, we were unable to collect these data which may lead to the bias of our results. On the contrary, all patients were thoroughly evaluated by an oncologist and pulmonologist before the start of chemotherapy treatment. Nevertheless, importantly our study characterizes an overt increase in the complications of pulmonary functions following cytotoxic chemotherapy, highlighting both the urgent vulnerability of patients with cancer during the pandemic as well as to determine the critical need of post-chemotherapy care including pulmonary rehabilitation.

## Conclusion

Based on our results, the evaluation of lung functions should probably be performed in all patients recovered from COVID-19 who require chemotherapy. Furthermore, we propose to follow these patients for a longer period of time for evidence of pulmonary rehabilitation to appropriately manage possible lung impairments occurring due to chemotherapy. A larger prospective study with an additional cohort of cancer patient without prior COVID-19 infection is thus necessary.

## Data Availability Statement

The raw data supporting the conclusions of this article are included in the article.

## Ethics Statement

The studies involving human participants were reviewed and approved by the Ethics Committee of Bangladesh Medical Research Council (BMDC) and Park View Hospital [Study # 2021-2023/62(1-20]. The patients/participants provided their written informed consent to participate in this study. Written informed consent was obtained from the individual(s) for the publication of any potentially identifiable images or data included in this article.

## Author Contributions

SSI has contributed to the conception and design of the study, contributed in data analysis, and overall supervision of the study. SSI, HY, WF, and MA-M contributed in writing the manuscript. SSI, AN, AK, MA-M, MI, HY, and WF have contributed to literature search, data collection, data interpretation, and clinical data inconsistencies. All authors reviewed the manuscript and have given final approval of the submitted version.

## Conflict of Interest

The authors declare that the research was conducted in the absence of any commercial or financial relationships that could be construed as a potential conflict of interest.

## Publisher's Note

All claims expressed in this article are solely those of the authors and do not necessarily represent those of their affiliated organizations, or those of the publisher, the editors and the reviewers. Any product that may be evaluated in this article, or claim that may be made by its manufacturer, is not guaranteed or endorsed by the publisher.
